# Impact of an electronic alert system for pediatric sepsis screening a tertiary hospital experience

**DOI:** 10.1038/s41598-022-16632-2

**Published:** 2022-07-20

**Authors:** Abdullah Alturki, Ayman Al-Eyadhy, Ali Alfayez, Abdulrahman Bendahmash, Fahad Aljofan, Fawaz Alanzi, Hadeel Alsubaie, Moath Alabdulsalam, Tareq Alayed, Tariq Alofisan, Afnan Alnajem

**Affiliations:** 1 Department of Pediatrics, King Faisal Specialist Hospital and Research Centre, Riyadh, Saudi Arabia; 2 Department of Pediatrics, College of Medicine, King Saud University, Riyadh, Saudi Arabia; 3 Maternity and Children’s Hospital, Alhasa, Saudi Arabia; 4 Research Center, King Faisal Specialist Hospital and Research Centre, Riyadh, Saudi Arabia

**Keywords:** Paediatrics, Disease-free survival

## Abstract

This study aimed to assess the potential impact of implementing an electronic alert system (EAS) for systemic inflammatory syndrome (SIRS) and sepsis in pediatric patients mortality. This retrospective study had a pre and post design. We enrolled patients aged ≤ 14 years who were diagnosed with sepsis/severe sepsis upon admission to the pediatric intensive care unit (PICU) of our tertiary hospital from January 2014 to December 2018. We implemented an EAS for the patients with SIRS/sepsis. The patients who met the inclusion criteria pre-EAS implementation comprised the control group, and the group post-EAS implementation was the experimental group. Mortality was the primary outcome, while length of stay (LOS) and mechanical ventilation in the first hour were the secondary outcomes. Of the 308 enrolled patients, 147 were in the pre-EAS group and 161 in the post-EAS group. In terms of mortality, 44 patients in the pre-EAS group and 28 in the post-EAS group died (*p* 0.011). The average LOS in the PICU was 7.9 days for the pre-EAS group and 6.8 days for the post-EAS group (*p* 0.442). Considering the EAS initiation time as the “zero time”, early recognition of SIRS and sepsis via the EAS led to faster treatment interventions in post-EAS group, which included fluid boluses with median (25th, 75th percentile) time of 107 (37, 218) min vs. 30 (11,112) min, *p* < 0.001) and time to initiate antimicrobial therapy median (25th, 75th percentile) of 170.5 (66,320) min vs. 131 (53,279) min, *p* 0.042). The difference in mechanical ventilation in the first hour of admission was not significant between the groups (25.17% vs. 24.22%, *p* 0.895). The implementation of the EAS resulted in a statistically significant reduction in the mortality rate among the patients admitted to the PICU in our study. An EAS can play an important role in saving lives and subsequent reduction in healthcare costs. Further enhancement of systematic screening is therefore highly recommended to improve the prognosis of pediatric SIRS and sepsis. The implementation of the EAS, warrants further validation in multicenter or national studies.

## Introduction

Despite several years of clinical and bench research, sepsis has remained the key cause of pediatric mortality in hospitals, and the only intervention that has reduced mortality is the early recognition of sepsis and the administration of antibiotics^1,2^. Pediatric early warning scores (PEWS) are used in various hospitals and have been integrated with the vital signs and laboratory values of patients’ electronic health records. Alert triggering thresholds have also been established^3^. Following the successful execution of PEWS, PEWS-identified patients have been found to have received expedited care services and, when indicated, have been transferred to the intensive care unit (ICU) considerably faster than non-PEWS patients. The provision of expedited care services translates into decreased mortality from sepsis^4^.

The electronic alert system (EAS) for systemic inflammatory response syndrome (SIRS) and sepsis alerts the care team to the deteriorating condition of patients with probable SIRS or sepsis. To achieve this, the EAS primarily evaluates the vital signs of patients and sends precise messages to the care team in instances where it forecasts or establishes a deterioration in the signs of sepsis. Based on such changes, the EAS then proposes the next steps that should be taken to avert a deterioration in the patient’s conditions^5^. The early diagnosis and employment of various therapeutic treatments are possible once patients with sepsis have been identified using the EAS^6^. The use of an EAS can significantly reduce the time before starting sepsis-linked treatment interventions^7^.

Due to the significant epidemiological burden of sepsis and a lack of clear consensus about a specific sepsis alert system that may aid in reducing the undesirable yet avoidable sequelae of sepsis, a hospital-wide SIRS/sepsis electronic alert system (EAS) was installed at King Faisal Specialist Hospital and Research Centre (KFSHRC) in Riyadh, Saudi Arabia. This study aimed to assess the impact of the use of the SIRS/sepsis EAS on mortality and length of stay (LOS) in a pediatric population admitted to the pediatric ICU (PICU) with SIRS or sepsis.

## Materials and methods

This retrospective study with a pre and post design was carried out at a tertiary hospital. We enrolled patients diagnosed with sepsis, severe sepsis, septicemia, or septic shock as a primary diagnosis upon admission to the PICU from January 2014 to December 2018. Children aged ≤ 14 years were included in this study if they had suspected or confirmed sepsis as per the definition of the Surviving Sepsis Campaign and were admitted to the PICU during the study period^8^. The causes of sepsis/severe sepsis were not relevant to their eligibility for the study. The eligible patients were identified by searching the electronic medical records and PICU database registry. Patients were excluded if their data were incomplete. The data were collected from the patients’ electronic health records and included demographic data and clinical profiles, which comprised vital signs upon admission, initial lab values, time of antibiotics initiation, need for inotropic support, and outcomes. The following outcomes were assessed: PICU LOS, survival and mortality rates, and severity of illness scores (PRISM, PIM) on admission to the PICU.

The patients were categorized into pre- and post-intervention groups, and the intervention was defined as the implementation of the SIRS/sepsis alerts via the EAS. In collaboration with a physician group, the healthcare information technology affairs at KFSHRC announced that the go-live date of the SIRS/sepsis alerts would be February 23, 2016. The cohort of patients who met the inclusion criteria during the period January 1, 2014, to February 22, 2016, were considered the pre-EAS (control) group, while those treated from February 23, 2016, to December 31, 2018, comprised the post-EAS (experimental) group. More detailed information related to the SIRS/sepsis alerts is provided in the supplementary file. We defined “the zero time” as the time documented in the system meeting the criteria for the alert initiation to trigger appropriate interventions. Therefore, when calculating the time to intervention, which may have included the first fluid bolus or the first dose of antibiotics, the zero time was based on the documented time of automated alert initiation in the system for the post-EAS group. For the pre-EAS group, the timing was based on the retrospective application and manual configuration of the same alert filter in the system to capture the zero time from electronic health records for these patients. For the variable "Mechanical ventilation in the first hour of admission", it was based on whether the patient required mechanical ventilation during the first hour after admission to the PICU irrespective to the zero time.

The data consisted of the patients’ electronic medical records and the associated data from the hospital database for quality assurance. In the statistical analysis, the categorical values were presented as percentages and the continuous variables as the mean (± standard deviation) for the parametric data and the median (25th, 75th percentile) for the nonparametric data. The chi-squared test and Fisher’s exact test were used to compare the proportions between the groups, Student’s *t*-test to compare the means, and the Mann–Whitney* U* test to compare the medians. To adjust for confounding variables, a multivariate logistic regression was conducted to consider the clinically relevant variables while avoiding possible colinearity. The model diagnostic metrics were calculated accordingly. A *p* value of 0.05 was considered significant, and all the statistical calculations were carried out using SPSS version 23.0 (IBM SPSS Statistics for Windows, Version 23.0. Armonk, NY: IBM Corp. IBM Corp). Given that improving mortality was the primary outcome of this study, our hypothesis was that the proportion of deaths in the post-EAS group would be 20–25% less compared to that in the pre-EAS group with the proviso that the pre-EAS sepsis mortality rate was 30%, the alpha was 5% (two-sided), and the power was 90%. The estimated sample size for the two-sample comparison was 161 for each group (pre- and post-EAS).

The study was approved by the institutional review board of the KFSHRC, and consent was waived due to the retrospective nature of the study. The data collection form included serial code numbers to ensure patient privacy. In accordance with KFSHRC policy, confidentiality was maintained by securing the forms in a locked cabinet with very limited access.

All methods were performed in accordance with the relevant guidelines and regulations of KFSHRC Institution Review Board.

## Results

The total number of enrolled patients with sepsis was 308 out of 4252 PICU admissions, which corresponds to a prevalence of 7.24% over the five-year period of the study. Of the 308 patients, 147 were in the pre-EAS group, and 161 were in the post-EAS group. The demographics and presentation variables between the two groups are shown in Table 1.

**Table 1:** Baseline demographic and clinical characteristics for the pre- and post-EAS groups.

Variables	Pre-EAS group n = 147 n (%)	Post-EAS group n = 161 n (%)	*p* value
**Gender**			0.36*
Male	80 (54.42)	96 (59.63)	
Female	67 (45.58)	65 (40.37)	
Age median (25th, 75th percentile) (years)	4.30(1.87,11.44)	5.56(0.79,10.68)	0.149^M^
**Age (years)**			
0–2	53 (36.05)	44 (27.33)	
2–7	39 (26.53)	32 (19.88)	0.0626*
8–12	35 (23.80)	54 (33.54)	
> 12	20 (13.60)	31 (19.25)	
Weight mean ± SD (kg)	17.21 ± 11.98	17.14 ± 13.2	0.962^t^
**Primary disease**			
Hematology/oncology	48 (32.65)	54 (33.54)	
Bone marrow transplant	30 (20.40)	41 (25.57)	
Immunodeficiency	11 (7.48)	2 (1.24)	
Genetic/metabolic	9 (6.12)	12 (7.45)	
Cardiac	24 (16.33)	11 (6.83)	0.0348*
Gastroenterology/hepatology	2 (1.36)	8 (4.97)	
Neurology	7 (4.76)	8 (4.97)	
Solid organ transplant	11 (7.48)	13 (8.07)	
Renal	2 (1.36)	6 (3.73)	
Endocrinology	2 (1.36)	4 (2.24)	
Other	1 (0.68)	2 (1.24)	
Pediatric risk of mortality score 3	14.06 ± 8.23	13.40 ± 9.66	0.523^t^

*EAS* electronic alert system, *SD* standard deviation.

The *p*-value was calculated using the indicated statistics: ^t^Student’s *t*-test, *chi-squared test, ^M^Mann–Whitney *U* test.

The median time before the first normal saline bolus administration to the patients in the pre-EAS group was 107 (37, 218) minutes compared to 30 (11,112) minutes for those in the post-EAS group (*p* < 0.001). The median time before the first dose of antimicrobials administration was 170.5 (66,320) minutes for the pre-EAS group, with 131 (53,279) minutes for the post-EAS group (*p* 0.042). The difference in mechanical ventilation in the first hour of admission was not significant between the pre- and post-EAS groups (25.17% vs. 24.22%, respectively; *p* 0.895), with an average total ventilation duration of 5.41 ± 7.71 and 5.36 ± 6.33 days, respectively (*p* 0.8988). In the pre-EAS group, 23 (15.6%) patients required high-frequency oscillation ventilation compared to 16 (9.9%) patients in the post-EAS group (*p* 0.6776). Inotropic support was needed for 112 (76.12%) of the patients in the pre-EAS group and in 112 (69.56%) of the patients in the post-EAS group (*p* 0.20).

The median number of organs affected during PICU stay in the pre-EAS group was 3(2, 3) and 2(1, 3) in the post-EAS group (p 0.0117). The average LOS in the PICU was 7.9 days in the pre-EAS group and 6.8 days in the post-EAS group (*p* 0.442).

In terms of survival, 44 (29.93%) patients died in the pre-EAS group whereas the total deaths in the post-EAS group was 28 (17.39%) patients (*p* 0.011). The details of the required interventions and outcomes are shown in Table 2. The multivariate logistic regression analysis for mortality as the dependent outcome revealed that younger age, a higher pediatric risk of mortality 3 (PRISM3) score, and being in the pre-EAS group were independent predictors of mortality, as shown in Table 3.

**Table 2:** Clinical interventions and outcomes comparison between the pre- and post-EAS groups.

Variables	Pre-EAS group n = 147 n (%)	Post-EAS group n = 161 n (%)	*p* value
**Interventions**			
Time to first dose of fluid bolus * (min.) Median (25^th^, 75^th^ percentile)	107 (37, 218)	30 (11,112)	< 0.001^M^
Time to first dose of antibiotics** (min.) Median (25th, 75th percentile)	170.5 (66,320)	131 (53,279)	0.042^M^
Inotropic support	112 (76.12)	112 (69.56)	0.20^#^
High-flow nasal oxygen	58 (39.46)	64 (39.75)	1^#^
Conventional mechanical ventilation first hour	37 (25.17)	39 (24.22)	0.895^#^
Conventional mechanical ventilation first 24 h	68 (46.58)	59 (36.64)	0.081^#^
High-frequency oscillatory ventilation	23 (15.65)	16 (9.94)	0.6776^#^
Mechanical ventilation duration (mean ± SD) (days)	5.41 ± 7.71	5.36 ± 6.33	0.8988^t^
Renal replacement therapy	10 (6.80)	8 (4.97)	0.6281^#^
**Outcomes**			
Number of organs affected Median (25th, 75th percentile)	3(2, 3)	2(1, 3)	0.0117^M^
PICU length of stay) (days) Median (25th, 75th percentile)	3.08 (1.49, 8.20)	3.71 (1.33, 9.11)	0.976^M^
Mortality	44 (29.93)	28 (17.39)	0.011^#^

*Patients who received fluid bolus in Pre-group were 96/147(65.3%), vs. 112/161(69.6%) in Post-group.

**Patients who received antibiotics in Pre-group 140/147(95.2%) vs. 153/161(95%) in the post–group.

*EAS* electronic alert system, *SD* standard deviation; The *p* value was calculated using the indicated statistics: ^t^Student’s *t*-test, ^#^Fisher’s exact test, ^M^Mann–Whitney *U* test.

**Table 3:** Multivariate logistic regression analysis for mortality as the dependent outcome.

	Odds ratio	*p*	Estimate	95% Confidence interval	
				Lower bound	Upper bound
Intercept	71.243	< 0.001	4.266	3.105	5.428
Age	1.129	0.002	0.122	0.045	0.198
PRISM 3 score	0.831	< 0.001	−0.185	−0.240	−0.130
Gender	0.612	0.174	−0.491	−1.199	0.217
Group (pre-EAS)	0.460	0.027	−0.776	−1.462	−0.089
Mechanical ventilation in first hour	0.497	0.062	−0.699	−1.432	0.034

*PRISM 3* Pediatric Risk of Mortality III; *EAS* electronic alert system.

Outcome level “Survived” was coded as class 1.

Model performance metrics: area under curve = 0.857, sensitivity = 0.940, specificity = 0.500, precision = 0.860.

## Discussion

In this study, we report a significant statistical and clinical reduction by almost half (i.e., from 29.93%% down to 17.39%) in mortality related to sepsis in pediatric cases admitted to the PICU upon implementation of an EAS for SIRS/sepsis in an in-hospital setting. This finding is mainly attributed to the successful integration of the early identification of potential sepsis cases and prompt management. As observed in the literature, among all the interventions aimed at improving survival in patients with sepsis, fast recognition and the timely initiation of therapy, including antimicrobial administration, are the most important components^1,2,9–13^. Different approaches have been investigated to address the timely implementation of the recommended management. These have ranged from increasing awareness, enhancing education, and delegation to specialized teams to the implementation of a timely protocol or pathway^6,14–18^. Compliance with the Surviving Sepsis Campaign performance bundles, which include early recognition and intervention, was associated with a 25% relative risk reduction in mortality in patients with severe sepsis and septic shock^19^. Nevertheless, these bundles and protocols may not be consistent in improving mortality due to compliance variability and concern regarding the identification of zero time in the timeline protocol^6,10^. Given that the diagnosis of potential sepsis cases is the cornerstone of therapeutic practice, a more structured and relatively objective approach is required to minimize human-related factors that could result in potential sepsis cases being missed. Such efforts led to the proposal of the PEWS system, which is widely accepted and has been validated in different settings with the potential benefit of identifying sepsis cases earlier and thus improving outcomes^1,9,20^. However, compliance with PEWS and integrated bundles requires reliable performance, high compliance, and regular audits, which could be challenging and resource demanding due to human factors^9,21,22^. The need for automated, effective, and timely screening tools for sepsis screening triggered further investment and investigations aimed at integrating health informatics and data analysis models^23,24^. The growing interest in machine learning and big data analysis fueled this innovative approach, which resulted in the development of automated decisions-aiding models and electronic alerts for potential sepsis cases^3,13,23,25–35^.

Many challenges and limitations can be encountered during electronic alert development, including differences in adult and pediatric parameters, the presentation timing, and the variable phenotypes of sepsis and the underpinning genomics subgroups^33,36–39^. For instance, compared to adults, pediatric sepsis recognition is challenging due to the low specificity of tachycardia and tachypnea and the relatively late manifestation of hypotension^38^. Furthermore, many reports have addressed the validation of EAS use in the emergency department, which could be different compared to in-hospital settings^25,26,32,39–42^. These differences highlight the importance of accumulating more evidence to validate and support the EAS approach.

Although the early recognition of sepsis is invaluable, it must be accompanied by and integrated with appropriate therapeutic interventions to realize its essential value. Through these efforts multifaceted improvements in sepsis diagnosis and management will become more legitimate^3,15,43^. In our study, the implementation of a sepsis EAS was associated with a shorter time to receive a fluid bolus and the first dose of antibiotics. The median time to receiving the antibiotics from the EAS trigger was shorter by 39 minutes (23%) and thus clinically relevant^2,44,45^. There were otherwise no changes in respiratory or hemodynamic support interventions. It is likely that the timely improvement in initial sepsis management with fluid and antibiotics, which was mediated by the sepsis EAS implementation, was associated with a reduction in mortality and LOS in PICU in our study. Similar findings were reported in a pediatric acute care setting where an analysis of the pre- and post-EAS implementation groups showed a reduction in both the 3-day sepsis-attributable mortality (2.53 vs. 0%] and 30-day mortality (3.8 vs. 1.3%)^30^. In adults, similar improvements in mortality and LOS outcomes have been associated with an EAS integrated with a multifaceted identification and intervention approach^3,15^. An improvement in sepsis mortality has also been reported following the implementation of an EAS in an adult emergency setting^2,13^.

Another important outcome improvement in our study was the reduction in the median number of organs affected in the post-EAS group. The marginal difference between the pre- and post-EAS groups with respect to the organs affected could explain the lack of a significant difference or change in respiratory, hemodynamic, and renal support between the groups. These clinical parameters likely did not reach statistical significance due to the relatively small sample used to detect differences in these variables as the study was powered to detect mortality differences as its primary outcome.

Although both groups in our study shared comparable basic characteristics in terms of gender, age, weight, and PRISM3 scores prior to enrollment in the study, the patients with hematology/oncology primary diseases were more prevalent than those with other diagnoses. This vulnerable population is likely to rapidly deteriorate when sepsis occurs, which may suggest that these patients could benefit from sepsis EAS implementation and that the cost-effectiveness ratio could become more favorable^22,46,47^.

The rate of admission was relatively slower in the post-group and subsequently, the duration of the enrollment was longer in order to fulfill the planned calculated sample size from each group. One of the possibilities include that implementation of the EAS helped to stabilize patients and reduce the number of severe cases of pediatric sepsis needing admission to the PICU. We could not confirm this possibility due to the requirement of more data relevant to cases activated by the EAS but did not require PICU admission, which was beyond our study scope and require different protocol and data set. The other possibility of potential confounder resulting in less cases and the perceived improvement in outcomes cannot be completely excluded, however, the severity-adjusted analysis with inclusion of PRISM score in the logistic regression model is expected to minimize such potential bias.

The impact of sepsis EAS could be further enhanced to include subcategories to decrease the signal’s noise for false or non-specific alerts. Identifying and focusing on specific alerts may help to improve the allocation of time and resources. For instance, Emmanuel et al. reported that the impact of alerts for potential device-associated infections resulted in more clinical interventions than less-specific alerts for central line-associated bloodstream infections, while neonatal sepsis alerts resulted in minimal interventions undertaken in response to alerts^27^.

The findings of this hospital-wide study are important as they show a significant improvement in the patient survival rate following the addition of an EAS, which is the strategic goal for all healthcare facilities in general and in pediatric sepsis in particular. However, these results need to be interpreted with due consideration of their limitations. The pre-and-post study design are well known for its limitation such as changes over the time that could potential confound the outcome. However, given the scope of the study and lack of remarkable changes in the definition of pediatric sepsis or major changes in the initial ward-based intervention in pediatric sepsis over the time span of the study, such study scope could benefit from this pre-and-post design while considering its limitation^48,49^. Different sepsis alert systems have been implemented by several healthcare institutes with the aim of improving the ability of healthcare practitioners to detect sepsis early and thus treat patients who meet the criteria for these alerts promptly. Notwithstanding, at present, no clear consensus exists on a single alert system as several barriers limit the wide implementation of a unified sepsis alert system. We believe that further studies testing such interventions are critical to reduce mortality in pediatric patients. In addition, further development of EASs are strongly recommended to improve the prognosis of pediatric sepsis as far as possible.

## Conclusion

In this study, the introduction of electronic SIRS and sepsis alerts resulted in a statistically significant reduction in the mortality rate among a pediatric population admitted to the PICU of our tertiary hospital. The early recognition of SIRS and sepsis facilitated by the EAS led to faster interventions, including fluid boluses and the initiation of antimicrobial therapy. Further research into, and the enhancement of, such alerts are essential to improve the prognosis of pediatric sepsis globally.

## Supplementary Information

Supplementary Information.

## Data Availability

The datasets during and/or analyzed during the current study available from the corresponding author on reasonable request.
