# Supplementary material for: Impact of an electronic alert system for pediatric sepsis screening a tertiary hospital experience
Source: Sci Rep. 2022 Jul 20;12:12436. doi: 10.1038/s41598-022-16632-2 (PMC9300636; doi:10.1038/s41598-022-16632-2)
Supplement: Supplementary file 1 — Supplementary Information. [file 41598_2022_16632_MOESM1_ESM.pdf]

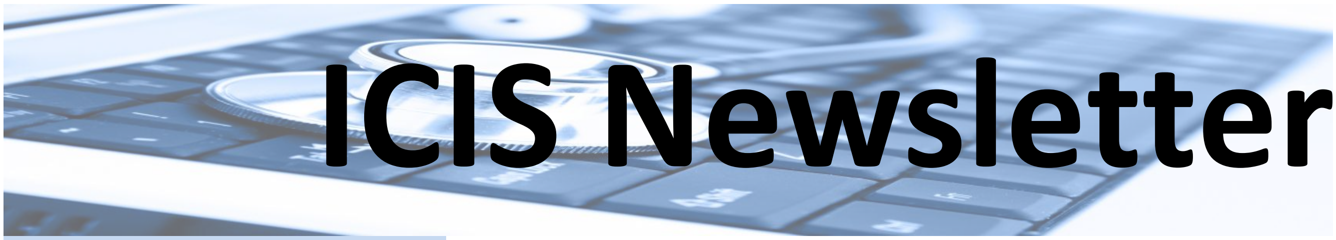

Go Live Date:

23rd Feb 2016 @ 0800

IPP:

MCO-MC-ADM-01-024

MCO-NA-NAA-01-021

Impacted End Users:

- **Inpatient & DEM Nursing (excluding Critical Care)**
- **Physicians**

Applications Affected:

- **PowerChart**
- **FirstNet**

Facilities Affected:

- **Riyadh**
- **CCC**
- **Jeddah**

Contacts:

Clinical Training Support Pagers

- **Riyadh (46244 / 46247)**
- **Jeddah (41085 / 41088)**

SIRS/Sepsis Alerts

Overview

HITA has received several departmental requests to introduce automation that will alert clinician's if/when patients start to develop signs & symptoms of SIRS/Sepsis. In collaboration with a physician group, HITA is announcing the Go-Live of SIRS/Sepsis alerts. This newsletter will aid clinicians in understanding the logic behind these alerts, how to handle alerts and the SIRS/Sepsis workflow.

Benefits

- Continuous silent screening of DEM and inpatient (non-critical care) populations.
- Alerts and triggers are customized to 8 age groups and generated as part of nursing documentation & posting of lab results.
- Automated alerts for patients meeting 3 or more SIRS/Sepsis criteria.
- Potential for earlier recognition and treatment of patients with Systemic Inflammatory Response Syndrome, or Sepsis.
- Prompts for additional lab orders to evaluate if a patient has sepsis.
- Documented history of all alerts, with criteria triggering the alert and the nurse/physician responses.
- Improved data collection and retrieval for statistical reports.

Highlights in Today's Issue

- **New!** - Overview of SIRS/Sepsis Alerts
- **New!** - The SIRS (Systemic Inflammatory Response Syndrome) Triggers
- **New!** - The Sepsis Triggers
- **New!** - SIRS/Sepsis Alerts
- **New!** - Addressing Orders, Tasks & Alerts
- **New!** - Reviewing Alert Histories
- **New!** - Changes to the FirstNet Tracking Board

1. Overview of SIRS/Sepsis Alerts

There are **8 (age specific) SIRS/Sepsis alerts** that silently and continuously evaluate predefined documented **vital signs** and **lab results**, in real time. The 8 alerts are age range based as follows;

- 0 days < 1 week of age
- 1 week < 1 month of age
- 1 < 12 months of age
- 1 < 2 years of age
- 2 < 6 years of age
- 6 < 13 years of age
- 13 < 18 years of age
- 18 < 150 years of age

For a SIRS alert to be triggered, it requires that 3 out of 6 criteria (in Section 2) are met. Although more than 3 criteria may have been met they may not be included in the alert. The alert will fire as soon as the rule receives the first 3 verified results that fall within the trigger range. The exclusion of results from an alert is more noticeable with lab results which can be authenticated at different times, as opposed to a set of vital signs which are generally documented under the same time frame, i.e. 0800. If only one or 2 triggers were met the system will retain those triggers/values as specified (in Section 2&3). For example, if a Temperature and Blood Glucose qualified as SIRS triggers, but those were the only 2 criteria met, the rule would retain both of these values for 30 hours. If at any time during the next 30 hours one or more additional SIRS criteria were met the alert would fire.

2. The SIRS (Systemic Inflammatory Response Syndrome)Triggers

Each of the 8 "age based" alerts (above) continuously evaluates 6 predefined triggers (below). If a patient meets 1 or 2 of the following SIRS criteria;

SIRS Triggers	Retention Period
**Heart Rate** - either Apical, Peripheral or Heart Rate Monitored	30 hours
**Respiratory Rate**	30 hours
**Temperature** - either Axillary, Oral, Rectal or Temperature Tympanic	30 hours
**WBC**	**48 hours**
**Band Man %** - will only be included if within range and the WBC was not a trigger	30 hours
☆ **Blood Glucose** - either Glucose, Random or POCT Blood Glucose	30 hours

A minimum of 3 positive SIRS triggers must be met, for a SIRS alert to fire.

the system will retain that met value as designated by the retention period. **If 3 triggers qualify than a SIRS alert will fire.** Each of the above SIRS triggers has an upper limit, lower limit or both. The trigger ranges vary based on the age specific alert, i.e. Heart Rate;

if patient is 0 days < 1 week of age, it will trigger if ≤ than 100, or ≥ than 180 bpm.
if patient is 2 < 6 years of age, heart rate will trigger only if ≥ 160 bpm.
if patient is 18 < 150 years of age, heart rate will only trigger if ≥ 120 bpm.

Blood Glucose ☆ result will be excluded by the rules, if the patient has an active **Problem** or **Diagnosis** related to **Diabetes Mellitus**, or is being administered oral or parenteral **hypoglycemic medications**.

3. The Sepsis Triggers

If a patient has qualified for any two of the SIRS criteria (in Section 2), or has an active SIRS alert, the above "age specific" rules (in Section 1) will continuously evaluate the 5 predefined

Sepsis triggers (below). **A Sepsis alert will fire as soon as any one** (of the below) **Sepsis triggers is met.**

Sepsis Triggers	Retention Period
**SBP** - Blood Pressure Systolic or Systolic Blood Pressure, Invasive	30 hours
**MAP** - Mean Arterial Pressure, Cuff or Mean Arterial Pressure, Invasive	30 hours
**Lactic Acid**	**12 hours**
**Bilirubin, Total**	30 hours
**Creatinine** - is evaluated based on a percentage increase from baseline	**72 hours**

A minimum of 2 positive SIRS triggers, or a SIRS alert **+ ONE Sepsis trigger** must be met, for a Sepsis alert to fire.

Creatinine result will be excluded by the rules, if the patient has an active **Problem** or **Diagnosis** that is **renal related**.

4. SIRS/Sepsis Alerts

Both SIRS/Sepsis screening and the alerts are passive. The screening occurs, and any applicable alerts appear without clinicians having to perform additional steps, or search for information. If a clinician is logged into any ICIS application and the (below) 3 criteria are met, any alert that fires will be viewable;

1) **Patient location -** the display of SIRS/Sepsis alerts is filtered by patient location (only DEM and Inpatient areas). Patients in all other locations will not qualify for SIRS/Sepsis screening and no alerts will fire.

2) **ICIS position -** only the following nursing positions can receive an alert;

   - Staff Nurse 1
   - Staff Nurse 2
   - Nurse / Scheduler
   - Supervising Nurse

3) **Patient Provider Relationships**

If the above 2 criteria are true, a SIRS/Sepsis alert will only display to clinicians that have an active Patient Provider Relationship with that patient. If there is no active PPR, no alert will be viewable, concurrently, if the clinician has active relationships with every patient on a unit, all alert(s) for all patient(s) on that unit will display. To prevent an overabundance of alerts, PPR relationships for these 4 positions will be expired after 3 days.

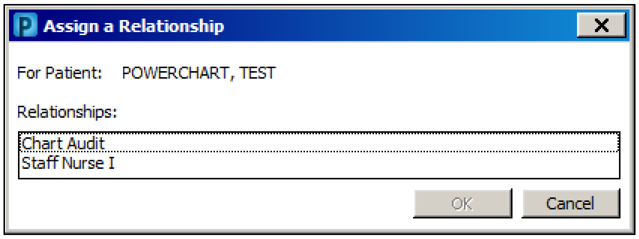

SIRS/Sepsis alert features the following components;

1) The Discern Notification title bar, and user ID.

2) An **overview of all alerts** that your position qualifies to see, based on patient location and active PPR's.

All **SIRS** alerts will have the subject **"Sepsis Screening Acute Care"** + patient name + Date/Time the alert fired.
All **Sepsis** alerts will have the subject **"Severe Sepsis Alert"** + patient name + Date/Time the alert fired.
New alerts will be bold, previously viewed alerts have non-bolded font.
Highlighting the required alert, will open that alert message in the view pane beneath.

3) The notification type (either **SIRS** or **Sepsis**).

4) Patient demographic data, with a **hyperlink** that opens the patient chart.

5) Recommendation to consider the following labs (if not recently completed - **SIRS only).** If the alert pertained to **Sepsis** the message would display as;

> The following information suggests that this patient may have sepsis. Please notify the physician immediately to ensure the patient is on the appropriate medication therapy. Early goal directed therapy is essential for the treatment of sepsis. Time dependent intervention may impact patient outcome.

6) **Hyperlink** that opens the patient chart.

7) A list of all of the qualifying criteria that pulled into the alert, Date/Time of result and the reference range for each trigger.

8) The minimize button to hide the Discern Notification window.

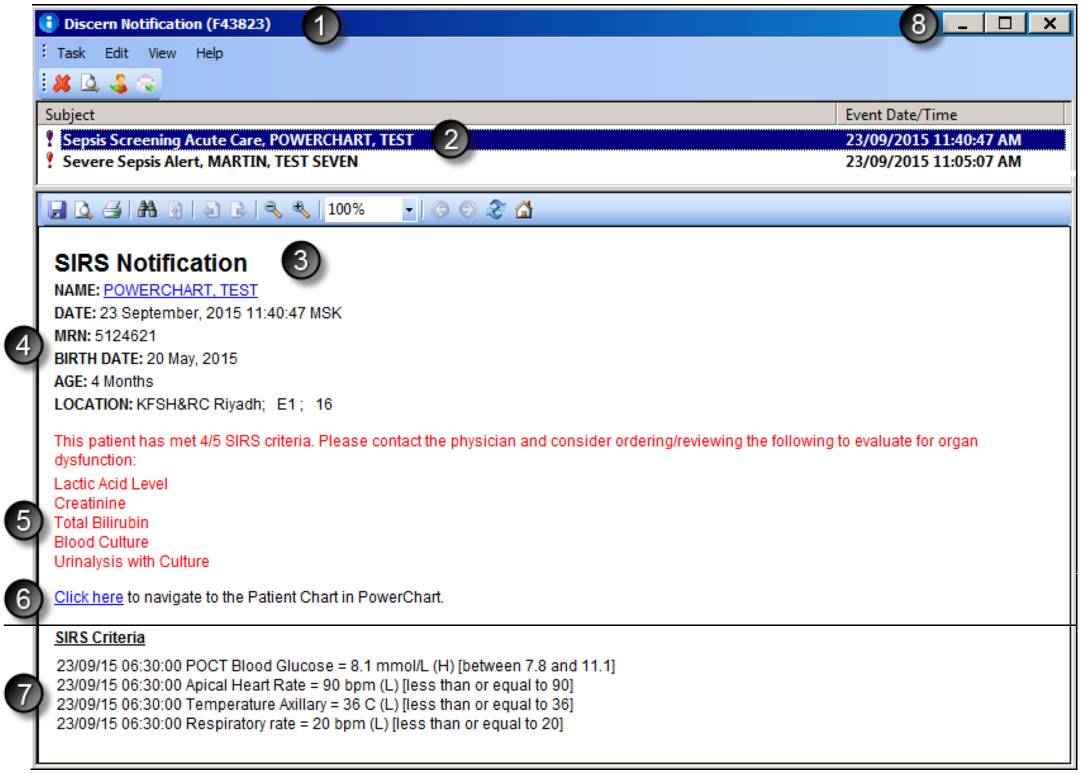

> **Note:** The Discern Notification window can't be closed, but rather only minimized to hide it. Allowing closure of this window would prevent clinicians from receiving alerts.

5. Addressing Orders, Tasks & Alerts

When an alert fires, it also auto-generates a corresponding SIRS or Sepsis order that displays under the clinical category of Patient Care. The order details display all SIRS/Sepsis criteria met. Follow the steps below to access the Task.

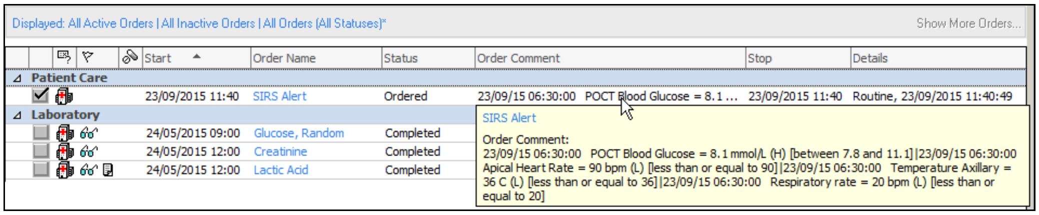

To doument the task;

1) Ensure the correct **date range** on the Task List.
2) Right click on the task.
3) Select **Chart Details** from the drop down menu.

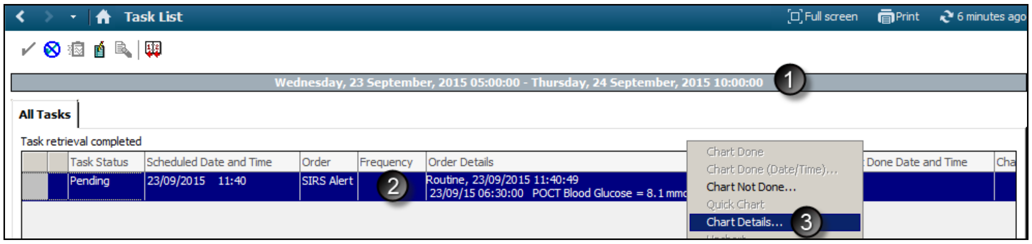

4) Enter the name of the **Physician Contacted**.
5) Enter the **Physician Response**.
6) Enter any pertinent remarks regarding the current alert or patient assessment in the **Nurse Notification Notes**.
7) Click the ☑ icon.

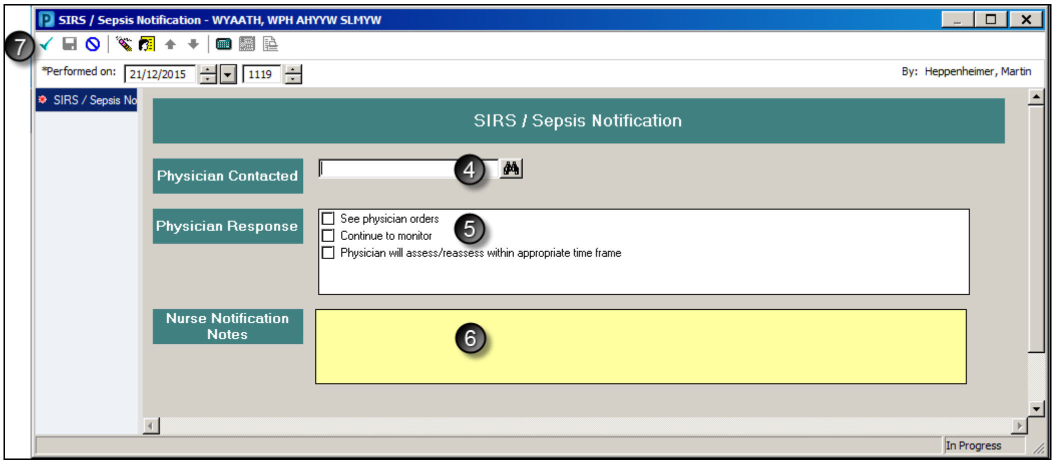

The task and order complete. Documentation will supress generation of a new alert [of the same type (SIRS/SIRS or Sepsis/Sepsis)] for a 24 hour period (starting from the time the alert triggered). A Sepsis alert could fire after a SIRS alert had been generated.

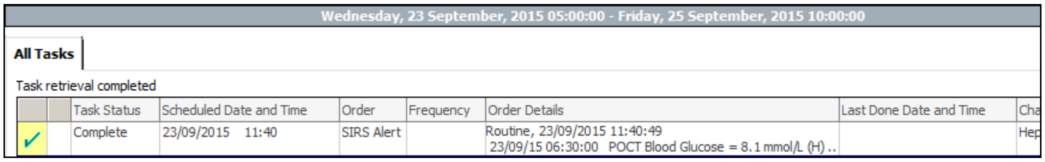

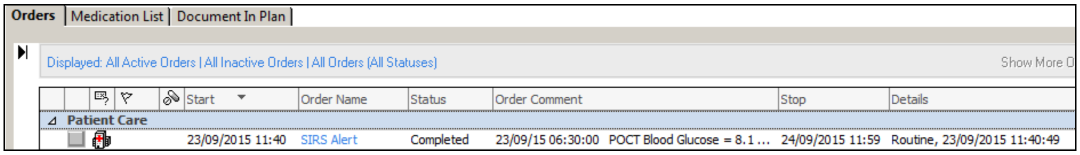

After documentation, both task and order will complete, however; **the alert will remain in the Discern Notification window** for 14 days. Each time the users logs into Cerner Millennium the SIRS/Sepsis alert will display.

Follow the below steps to open the Discern Notification window and remove the historic alert.

1) Click on the 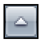 button in the bottom right corner of the Task Bar (near the Date/Time display).
2) Click on the flashing 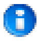 or 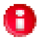 icon.

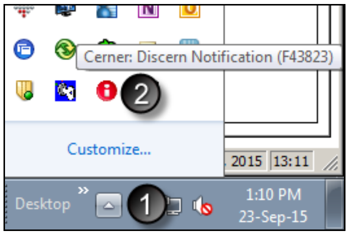

3) The **Discern Notification** window opens.
4) Right click on the completed alert.
5) Select **Delete Message** from the drop down list.
6) Click the 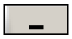 minimize icon to hide the Discern Notification window.

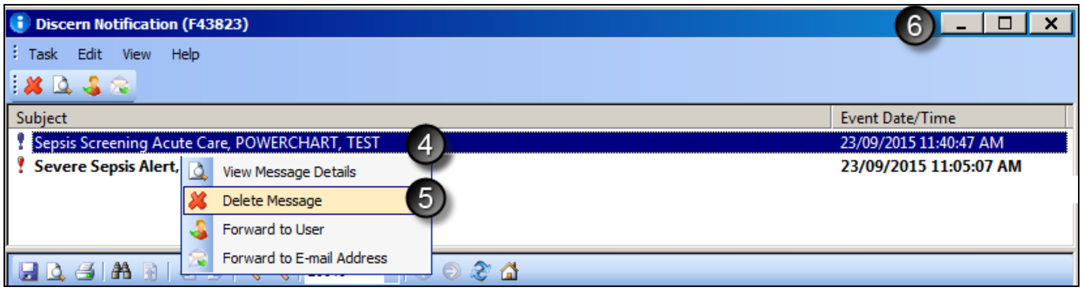

6. Reviewing Alert Histories

Historic alerts & documentation can be viewed from PowerOrders, Form Browser and the Flowsheet. Within PowerOrders, both SIRS and Sepsis orders display beneath the Clinical Category of Patient Care.

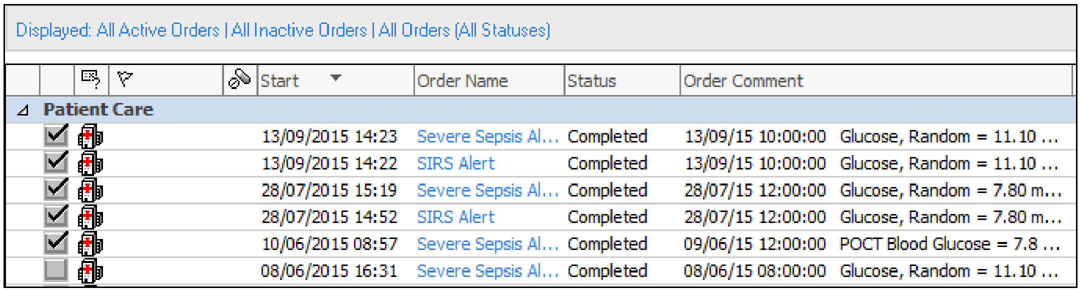

Displayed: All Active Orders | All Inactive Orders | All Orders (All Statuses)

Start	Order Name	Status	Order Comment
**Patient Care**			
13/09/2015 14:23	Severe Sepsis Al...	Completed	13/09/15 10:00:00 Glucose, Random = 11.10 ...
13/09/2015 14:22	SIRS Alert	Completed	13/09/15 10:00:00 Glucose, Random = 11.10 ...
28/07/2015 15:19	Severe Sepsis Al...	Completed	28/07/15 12:00:00 Glucose, Random = 7.80 m...
28/07/2015 14:52	SIRS Alert	Completed	28/07/15 12:00:00 Glucose, Random = 7.80 m...
10/06/2015 08:57	Severe Sepsis Al...	Completed	09/06/15 12:00:00 POCT Blood Glucose = 7.8 ...
08/06/2015 16:31	Severe Sepsis Al...	Completed	08/06/15 08:00:00 Glucose, Random = 11.10 ...

To access old documentation;

1) Select **From Browser** from the Menu.
2) Right click on the appropriate form.
3) Select **View** from the drop down menu.

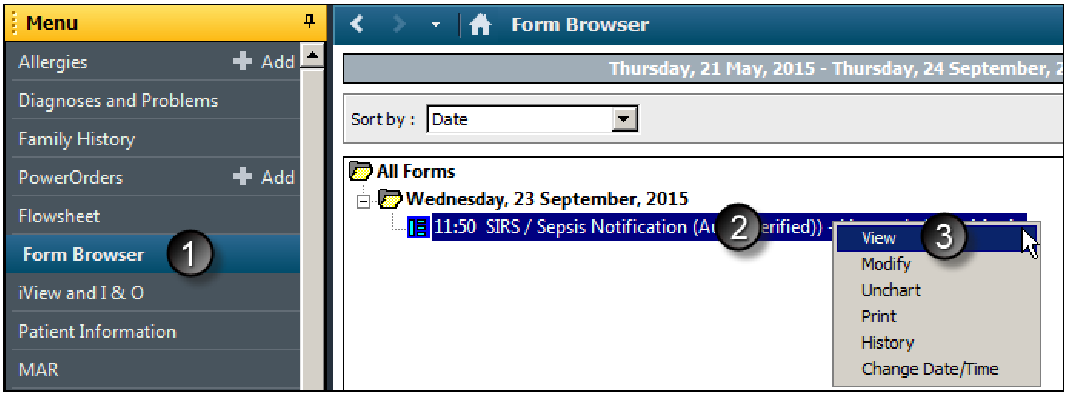

To access SIRS/Sepsis data from the Flowsheet;

4) Select **Flowsheet** from the Menu.
5) Click on the ▼ of the Flowsheet filter.
6) Select the **Early Warning Alerts** filter.

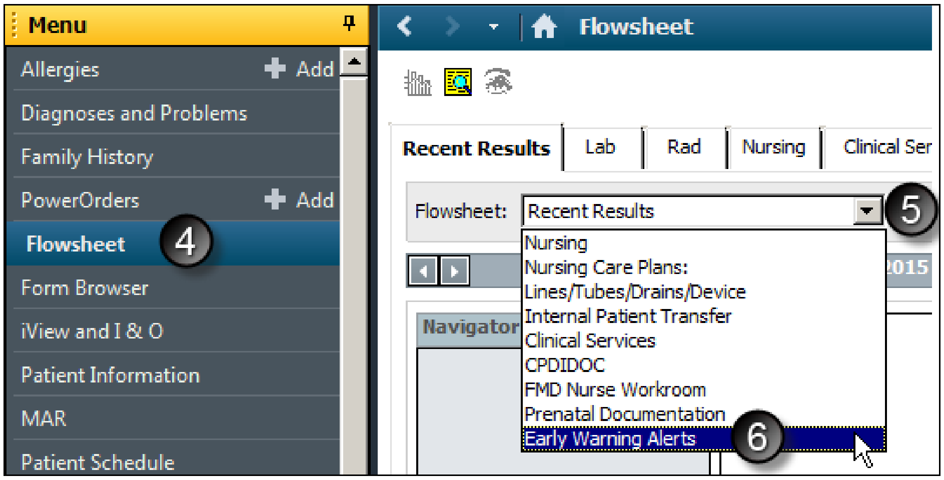

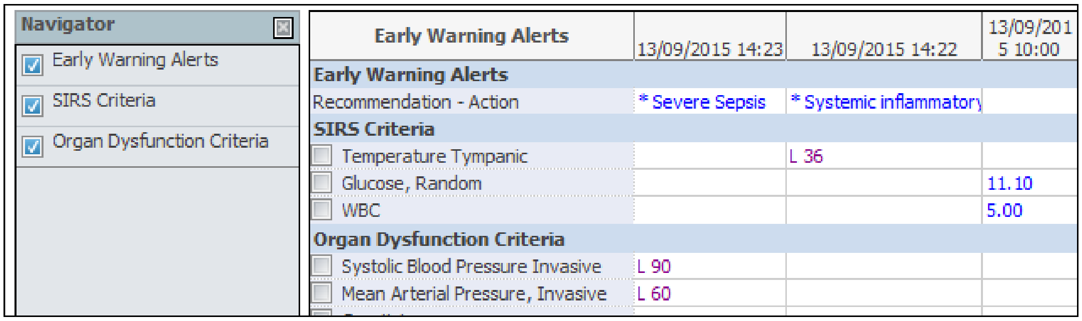

The **Early Warning Alerts** filter displays the type of alert (SIRS/Sepsis) and all triggers with correspond values that qualified for that alert.

7. Changes to the FirstNet Tracking Board

A new column “**Sepsis Alerts**” has been added to the FirstNet Tracking Board. If either a SIRS or Sepsis alert is triggered on any patient in DEM the following icons will display;

SIRS 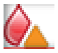

Sepsis 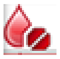

Hovering over the icon will display a tool tip with the order details.

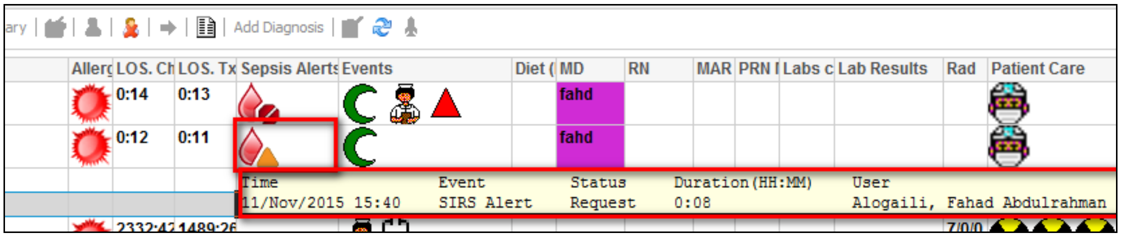

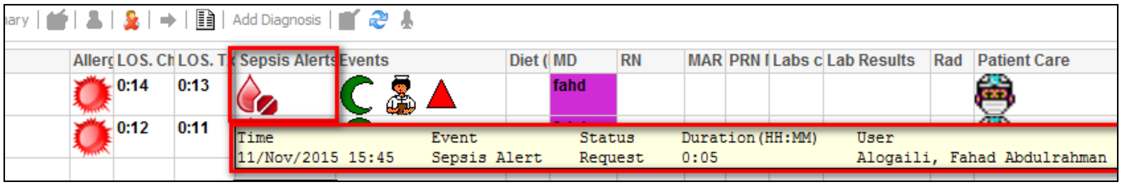

Access the SIRS / Sepsis Notification PowerForm thru the usual manner and document all applicable aspects of the alert as discussed with the physician. All other SIRS/Sepsis functionality (outside of the Tracking Shell) is equivalent to PowerChart and is covered in the above sections.
